# Designed physical activities targeting social skills in preschoolers: a meta-analysis

**DOI:** 10.3389/fpsyg.2025.1585415

**Published:** 2025-09-01

**Authors:** Yuxin Yuan, Wen Liu, Jingyao Yi, Xiaofen Li

**Affiliations:** School of Art, Beijing Sport University, Beijing, China

**Keywords:** designed physical activity, social skills, preschool children, meta-analysis, preschooler, physical activity

## Abstract

**Objectives:**

To comprehensively evaluate the impact of Designed Physical Activities (DPA) on the social skills of preschoolers, as well as its specific subdomains.

**Methods:**

We obtained data from Web of Science, EBSCO, cochrane library, PubMed, PsycInfo, China National Knowledge Infrastructure, WanfangData, and VIP Data from the establishment of each databases to June, 21, 2024. Two researchers independently assessed the quality of the study using the Cochrane risk of the bias assessment tool. Meta-analysis was performed when data were available, with further subgroup analysis, using Review Manager 5.4, and sensitivity analysis was performed using Stata software 15.1.

**Results:**

Search terms yielded 7,074 articles, of which 14 fulfilled the inclusion criteria. Results showed that DPA had beneficial effects for social skills [SMD = 0.63, *p* < 0.0001], and insignificant positive effects for emotional skill [SMD = 1.86, *p* = 0.08]. Subgroup analysis indicated that interventions with both music and tools, and without music or tools caused positive effects, while those with only music or only tools did not. Considerable outcomes were achieved regardless of the frequencies of the intervention. Interventions of 12 weeks and had a significant effect in promoting preschoolers’ social skills. Notably, the outcomes reported by parents were significantly higher than that reported by teachers and tested by children.

**Conclusion:**

DPA can significantly improve social skills and emotional skill in preschoolers. Nevertheless, it is imperative to conduct further trials with meticulous and rigorous study designs in order to furnish more definitive evidence in the foreseeable future.

## 1 Introduction

Social skills are the skills that help people interact with others, express their own emotions, understand others, and communicate with others both verbally and non-verbally in an appropriate way based on social principles (Takahashi et al., 2015; Ba, 2022). The definition of social skills covers lots of aspects, making psychologists still struggling on the accurate definition (Ogden, 2003). Different understandings lead to different opinions on the sub-domains. Gresham et al. (2011) categorized social skills into 5 sub-domains: cooperation, assertion, self-control, responsibility, and empathy, while Jurevičienė et al. (2012) considered social skills as interaction, communication, participation, emotional, and social cognition.

An increasing number of psychologists have realized the importance of social skills. Social skills are believed as a reliable measure of the quality of social behavior (Pečjak et al., 2009). According to previous researches, social skills enable children to have better preparation for school, helps them build better peer relationships and have positive interpersonal communication, and improves adaption to new environment that they may face in the next stage of life (Aksoy and Baran, 2010; Ziv, 2013). Children’s social skills are largely influencing their holistic growth and hole life, benefiting them not only short-termly but also long-termly (Gulay and Akman, 2009).

Psychologists have already reached on consensus that preschool age is a vital and sensitive period for individuals to develop sufficient social skills (Kramer et al., 2010; Moore et al., 2015). The social skills children in this period acquire could help them more efficient in expressing and understanding, and to get better profound development including elementary school adaption, quality of life, academic performance, and problem-solving (Arslan et al., 2011; Hosokawa and Katsura, 2017; Odom et al., 2008). Insufficient social skills could cause bad influences on children’s cognition, emotion, and behavior, which may lead to barriers in mental and psychological development of individuals. According to the results of previous studies, children with deficits in social skills may have more disorders in internalizing and externalizing behavior, poorer academic performance, more inappropriate reconciliation and adjustment, and worse relationships with parents, peers, and teachers, impeding in further development and leading to potential severe psychological issues (Karimi et al., 2010; King and Boardman, 2006; Lodder et al., 2016; Maleki et al., 2019a, 2019b; McClelland and Morrison, 2003; McClelland et al., 2000; Whitted, 2011; Ziv, 2013).

The World Health Organization (WHO) have estimated that there is at least 1 child suffering from ASD out of 100 children (World Health Organization, 2023), indicating that the prevalence of poor social skills and bad social relationships and behavior should be treated immediately and not be ignored. Among the interventions targeting social skills, physical activity has garnered the attention of researchers due to its fewer side effects compared to pharmacological treatments and its potential as a preventive measure. Therefore, researchers have designed physical activity (DPA) based on the characteristics and needs of children’s physical and mental development. DPA refers to physical activities specifically designed for preschool children, according to their development in the aspects of motor, cognition, social, and emotion, aiming to promote these aspects. The characteristics of DPA are as follows: (1) aiming to promote the comprehensive development of children, (2) designed with safety, fun, appropriateness, diversity, and educational value, (3) implemented with planning, guidance and interaction, systematicity and coherence, as well as assessability. The specific content of DPA can be diverse, but it must be interesting for young children, such as dancing, gymnastics, and various games. Depending on the specific DPA content and tasks, teachers may use different equipment and music when organizing activities, and set different durations and frequencies. However, DPA caused mixed results. The interventions such as peer-mediated interventions (Martinez et al., 2021; Zhang et al., 2022), structured play activities (Loukatari et al., 2019; Vidoni, 2007), group activities (Bonvin et al., 2013; Takala et al., 2011), cross-cultural physical activities (Tsangaridou et al., 2014), Preschool PATHS (Domitrovich et al., 2007), MyTeachingPartner Professional (Hamre et al., 2012) have shown positive effects (Gresham, 2001), while other interventions have reported limited or even negative effects (Lindsay and Dockrell, 2012).

The design of physical activity interventions plays a significant role in influencing the outcomes. Previous studies have shown that in addition to individual factors in children (such as gender and age) that can affect the results, differences in the design of intervention programs also have an impact on the outcomes. For example, Xu et al. (2022) argued that the frequency of the intervention can significantly influence the results. Lakes et al. (2019) and Du et al. (2024) emphasized the impact of combining music with physical activity on the outcomes. Among numerous factors that influence the outcome of the interventions, our study focused on the moderate effects of the use of “music and prop integration.” Previous studies have indicated that musical activities could positively impact children’s social skill development (Gilbert, 1992). For both adults and children, group singing, clapping, and playing instruments can enhance intimacy and trust, and boost behaviors like helping, cooperating, and coordinating that promote social skills (Wan and Zhu, 2021). Thus, introducing music into physical activities may further enhance the positive effect of interventions on children’s social skills, yet this difference remains to be verified through systematic research. On the other hand, the use of props (such as balls, ribbons, hula hoops, etc.) may increase the sensory stimulation and interaction quality of activities, providing more scenarios for role-playing and cooperative communication for children, which in turn may positively impact their social skill development.

Early research has made significant discoveries. Yet, there’s still a lack of systematic integration of prior studies to summarize early findings and compare the strengths and limitations of these research designs. Rather than simply stating that some studies were effective and others were not, integrating study outcomes and analyzing the process constitutes research problems. Therefore, we aimed to conduct a meta-analysis to integrate the outcomes of previous research according to our criteria, and describe and determine the effect of the DPA on social skills in preschool children, as well as a subgroup analysis to determine the influencing factors, aiming to support the social development of preschool children, and to offer evidence-based information and recommendations. This holds great significance for the feasibility and scalability of the intervention programs.

## 2 Methods

The selection procedure, study identification, and critical appraisal of the research studies were conducted according to the checklist presented in the Preferred Reporting Items for Systematic Reviews and Meta-analyses (PRISMA) statement (Page et al., 2021). The present study has been registered on PROSPERO (ID: CRD42024586359).

### 2.1 Search strategy

We performed a comprehensive search in the following eight databases: Web of Science, EBSCO, cochrane library, PubMed, PsycInfo, China National Knowledge Infrastructure, WanfangData, and VIP Data. We searched the databases from the establishment of each databases to June, 21, 2024. We used the PICOS framework to identify the keywords and also used the Boolean search method. Taking Web of Science as an example, the search formula was as follows: TS = ((exercis* OR physical activit* OR exercise training OR physical training OR aerobic exercise OR game OR sport) AND (preschool child*) AND (social skill* OR social abilit* OR social competence OR interaction skill* OR communication skill* OR participation skill* OR emotional skill* OR social cognition skill*)).

### 2.2 Inclusion and exclusion criteria

Trials examining the effect of DPA on social skills and its sub-domains in preschoolers were selected following PICOS (Participants, Intervention, Comparison, Outcomes, and Study design) inclusion criteria: (1) participants aged 3 ~ 6 years old of any sex, (2) intervention with DPA, including any types of ball games, dance exercises, yoga exercises, etc., without restriction regarding the control method; (3) comparison with any type of control means; (4) outcome with at least one validated quantitative generic rating scale of social skills or its sub-domain; (5) controlled designs, either parallel groups or crossover, with or without randomization or blinding.

The studies marked as review articles, retrospective studies, case reports or series, protocols, editorials, notes, and commentaries were not included. Only articles with full data access and written in English, Chinese, or Korean were considered eligible for inclusion.

### 2.3 Study selection

Two researchers (YY and WL) independently screened all the included studies by reading the title, abstract, and, if necessary, the main text of the article to determine whether the studies were eligible for review. The reasons for the ineligibility of a study were recorded. Subsequently, the two researchers had a discussion to reach a consensus. The third researcher (XL) intervened in the discussion in the case of a disagreement. Any discrepancies between the researchers were resolved by discussion.

### 2.4 Methodological quality appraisal

The Cochrane Collaboration Risk of Bias tool was used to assess the quality of the included studies. Based on the Cochrane Handbook, the evaluation included random sequence generation, allocation concealment, blinding of outcome assessment, incomplete outcome data, selective reporting, and other biases. All aspects of the included studies were assessed as low, unclear, or high risk of bias (Higgins, et al., 2019).

### 2.5 Data extraction and synthesis

All the relevant information about each study was extracted by two researchers (YY and JY) independently using a self-designed standardized form, which included basic information (the first author, the year of publication, and the region) and experimental information (such as participants, intervention characteristics, and outcome measures). Data missing was handled by contacting the authors of the included studies. Any discrepancy was resolved by discussion.

Meta-analysis was performed using Review Manager 5.4, and a leave-one-out analysis was performed to assess the sensitivity using Stata 15.1. The summary statistics for each outcome were the mean change from baseline and standard deviations (SD) of the mean change. The mean change in each group was obtained by subtracting the final mean from the baseline mean. The SD of mean change was computed in line with Follmann et al. (1992), which assumed a conservative correlation coefficient of 0.5. The standardized mean difference (SMD) and 95% confidence interval (CI) were calculated for the summary effect of continuous data. Results were considered significant when the CI did not include zero.

Heterogeneity was determined by the Cochrane Q statistic and the *I^2^* statistic. Cochrane Q statistic was used to test the heterogeneity, and the *I^2^* statistic was used to evaluate its value. The value of the *I^2^* statistic of 25, 50, and 75% indicated that the degree of heterogeneity between studies is low, moderate, and high (Higgins et al., 2003). The fixed-effects model was used when the *I^2^* < 50% and the random-effects model was used when the *I^2^* > 50%. The studies with a greater variance in their effect size estimate contributed less to the summary effect. When high heterogeneity exists, we conducted subgroup analysis based on several variables. We used Egger’s regression asymmetry test to test the publication bias (Egger et al., 1997). We conducted a leave-one-out analysis to observe the stability of the results.

## 3 Results

### 3.1 Research process

**Figure 1:**
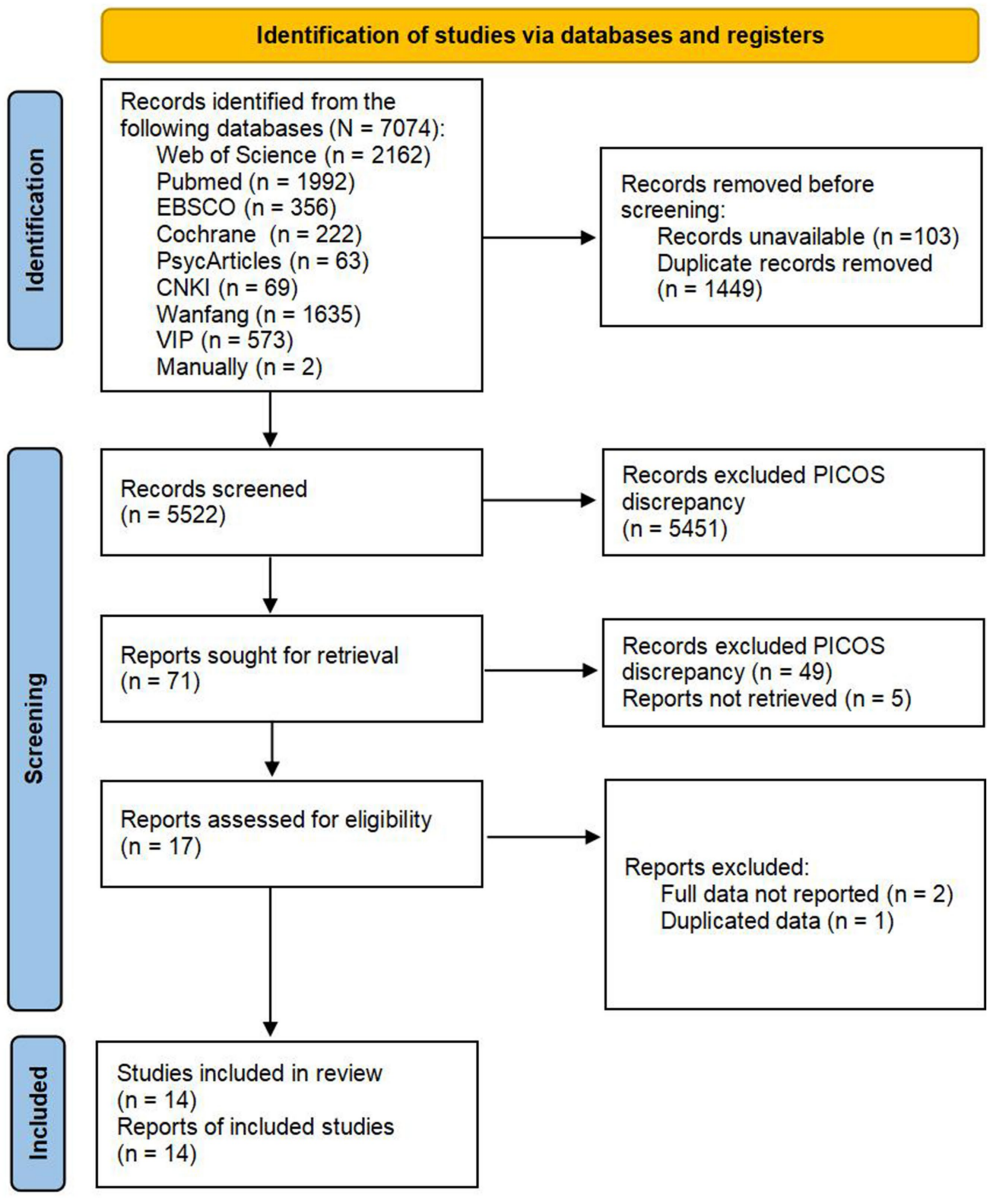
Literature selection flow diagram.

The results of the search process are shown in Figure 1. The bibliographical search yielded 7,074 citations, including 2 citations searched manually, published from the establishment of the eight databases mentioned above to June, 21, 2024. 103 citations were not included due to no availability, and 1,449 records were removed due to duplication. After reading the title and abstract, 5,451 citations were excluded due to PICOS discrepancies. The full text of the remaining studies (*n* = 71) was assessed for eligibility based on the inclusion criteria. A total of 14 studies were considered eligible for review.

### 3.2 Risk of bias assessment

**Figure 2:**
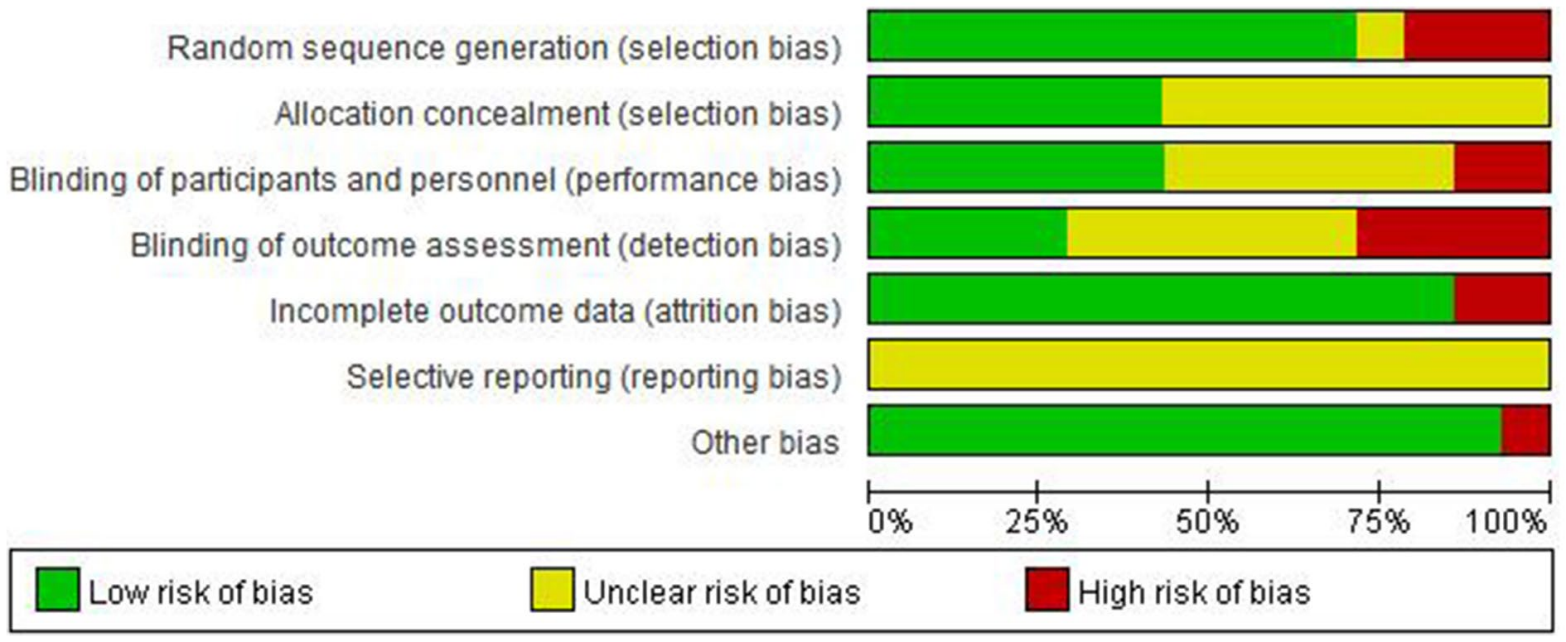
Percentage of biased items included.

**Figure 3:**
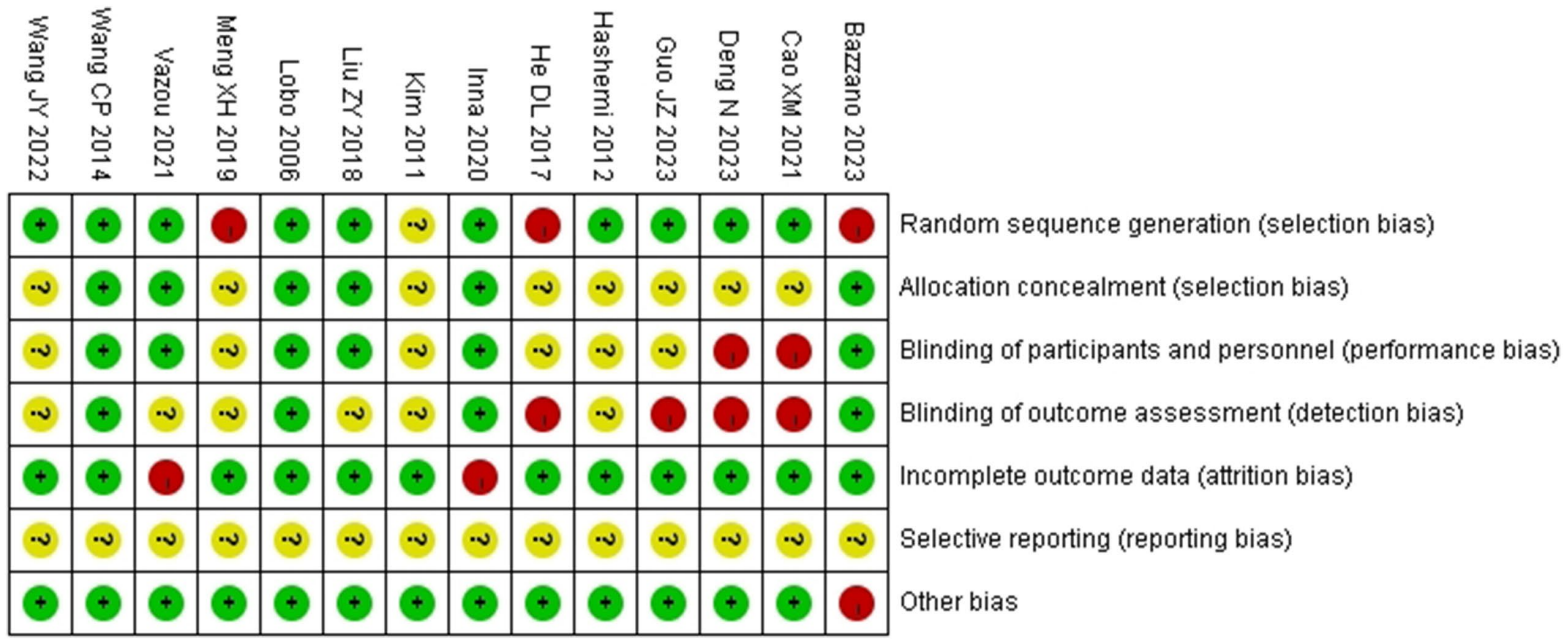
Risk of bias assessment results of included studies.

We performed a Cochrane risk of bias assessment for each study. The full results are shown in Figures 2, 3. Overall, the included studies had good methodological quality, with four studies rated with strong quality, seven studies with moderate quality, and three studies with low quality.

### 3.3 Study characteristics

The characteristics of the study are shown in Tables 1, 2. The included studies were performed in China (*n* = 8) (Cao, 2021; Deng N., 2023; Guo, 2023; He, 2017; Liu, 2018; Meng, 2019; Wang, 2014; Wang J., 2022), the USA (*n* = 3) (Bazzano et al., 2023; Lobo and Winsler, 2006; Vazou and Mavilidi, 2021), Israel (*n* = 1) (Kats Gold et al., 2021), Iran (*n* = 1) (Hashemi et al., 2012), and Korea (*n* = 1) (Kim et al., 2011). 1 study was identified as historical control trail, 10 studies were identified as randomized controlled trail, and 3 studies were identified as controlled trail. A total of 1,857 participants were covered in this review, of which 704 were included in the intervention groups and 1,153 in the control groups. The sample size of a single study ranged from 40 to 701. All the participants were preschool children, aged between 3 and 6 years. Eleven studies reported the percentage of participants’ gender, while 1 study only reported ‘approximately 50%’, and 2 studies did not report it. The intervention of the included studies were traditional physical games (1), creative dance (1), contextualized games (with/without balls) (2), rule-based sport games (1), traditional ethnic sports games (1), basketball games (1), football games (1), physical group games (1), yoga and mindfulness (1), gymnastics (2), “I Can Succeed for Preschools” program (1), and “Move for Thought (M4T) preK-K” program (1). The interventions were classified into with both music and tools (*n* = 7), with only music (*n* = 1), with only tools (*n* = 3), and without music or tools (*n* = 1). The intervention was for 10–60 min, 1–7 times per week, and lasted for 6–32 weeks. The measurement was completed by teacher (*n* = 6), parents (*n* = 8), and children (*n* = 3). Only 1 study reported the follow-up data.

**Table 1:** General characteristics of the included studies.

Study	Sample size (male %)	Age	Study design	Intervention	Control condition	Type	Intervention dose	Measured by
Bazzano et al. (2023), USA	I = 122 (50) C = 579 (50)	3 ~ 5	Historical control	Yoga and mindfulness	/	Music No tools	20 min/time 1 times/week 32 week	Teacher
Hashemi et al. (2012), Iran	I = 30 (50) C = 30 (50)	3 ~ 5	RCT	Gymnastics program	Usual activities	No music No tools	60 min/time 1 times/week 2 week	Parents
Kats Gold et al. (2021), Israel	I = 49 (38.8) C = 43 (55.8)	5 ~ 6	RCT	I Can Succeed for Preschool (ICS-PS)	Treatment as usual	No music With tools	/ / 28 week	Teacher, children
Kim et al. (2011), Korea	I = 24 (50) C = 24 (58.3)	4 ~ 5	CT	Traditional physical games	Usual physical activities	No music With tools	25 ~ 30 min/time 2 times/week 6 week	Parents
Lobo and Winsler (2006), USA	I = 19 (/) C = 21 (/)	3 ~ 6	RCT	Creative dance/movement	Attention control	Music With tools	35 min/time 2 times/week 8 week	Teacher, Parents
Vazou and Mavilidi (2021), USA	I = 130 (51.5) C = 129 (53.7)	3 ~ 5	RCT	Move for Thought (M4T) preK-K program	Usual physical education	Music With tools	10 ~ 20 min/time 7 times/week 8 week	Teacher
Cao (2021), China	I = 26 (53.8) C = 26 (57.7)	5 ~ 6	RCT	Situationalized Ball Game Course	Usual physical activities	No music With tools	40 min/time 2 times/week 10 week	Teacher, Parents
Deng N. (2023), China	I = 29 (55.2) C = 26 (50)	5 ~ 6	RCT	Regular Sports Game	Usual physical games	Music With tools	30 min/time 2 times/week 12 week	Parents
Guo (2023), China	I = 20 (50) C = 20 (50)	5 ~ 6	RCT	Traditional physical games	Usual physical education	Music With tools	20 ~ 30 min/time 3 times/week 12 week	Children
He (2017), China	I = 120 (60) C = 120 (60)	6	CT	Basketball Game Course for Preschoolers	Free play	Music With tools	30 min/time 5 times/week 12 week	Parents
Liu (2018), China	I = 24 (62.5) C = 24 (58.3)	4 ~ 6	RCT	Physical Group Games	Usual physical education	No music With tools	30 min/time 2 times/week 12 week	Teacher
Meng (2019), China	I = 25 (60) C = 25 (60)	6	CT	Football Course for Preschoolers	Usual physical education	Music With tools	30 min/time 5 times/week 12 week	Parents
Wang (2014), China	I = 30 (/) C = 30 (/)	5 ~ 6	RCT	Basic Gymnastics for Preschoolers	Free play	Music Without tools	40 min/time 6 times/week 15 week	Children
Wang J. (2022), China	I = 56 (46.4) C = 56 (42.9)	5 ~ 6	RCT	Situationalized Physical Game Course	Usual physical education	Music With tools	30 min/time 2 times/week 12 week	Parents

I, intervention group; C, control group; RCT, Random controlled trial; CT, controlled trail; /, not reported.

**Table 2:** Summary of outcome measures.

Outcome	Measurement tool	Study
Social skills	SSRS, Social skills rating system	Deng N. (2023); Kats Gold et al. (2021); Vazou and Mavilidi (2021)
	Iowa social competence scale, preschool form	Kim et al. (2011)
	SCBE, social competence behavior evaluation: preschool edition	Lobo and Winsler (2006)
	Passport for life	Guo (2023)
	Early Childhood Psychological Indicators Scale	He (2017); Meng (2019); Wang J. (2022)
	Questionnaire for Preschoolers’ Peer Interaction Skills	Liu (2018)
	Early Childhood Mental Health Questionnaire	Wang (2014)
	PKBS-2, Preschool and Kindergarten Behavior Scales	Hashemi et al. (2012)
Emotion	DECA-P2, The Devereux Early Childhood Assessment for Preschoolers, Second Edition	Bazzano et al. (2023)
	EMT, Emotion Matching Task	Kats Gold et al. (2021)
	CEAS-P, Children’s Emotional Adjustment Scale–Preschool Version	Cao (2021)

### 3.4 The overall analysis of the effects of DPA

#### 3.4.1 The effects of DPA on preschoolers’ social skills score

**Figure 4:**
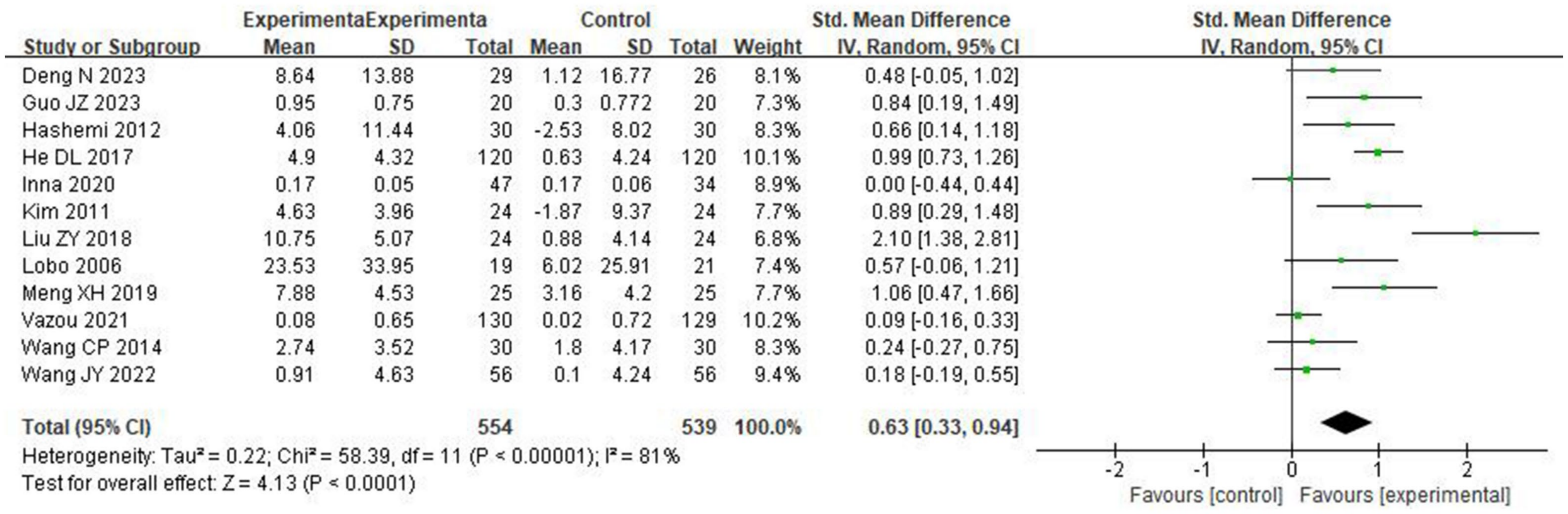
The effects of DPA on preschoolers’ social skills score.

A total of 12 studies determined the effects of DPA on the social skills testing score of preschool children. The score of social skills test is positively correlated with the social skills of the subject, and the weight of each study was determined (Figure 4). The result showed significant differences between groups [SMD = 0.63, 95% CI (0.33, 0.94), *p* < 0.0001], with high and significant heterogeneity (*I^2^* = 81% and Chi^2^ *p* < 0.00001).

#### 3.4.2 The effects of DPA on preschoolers’ emotional skill score

**Figure 5:**
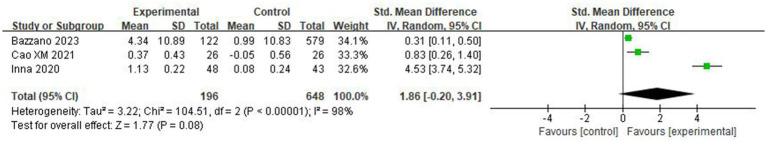
The effects of DPA on preschoolers’ emotion score.

A total of 3 studies determined the effects of DPA on the emotional skill score of preschool children. The score of emotion is positively correlated with the emotional skill of the subject, and the weight of each study was determined as Figure 5. The result showed insignificant differences between groups [SMD = 1.86, 95% CI (−0.20, 3.91), *p* = 0.08], with high and significant heterogeneity (*I^2^* = 98% and Chi^2^ *p* < 0.00001).

### 3.5 Subgroup analysis

In the intervention research on children, both the internal factors of children and the differences in intervention design can lead to differences in the development of children’s social skills. However, although gender and age are important factors, this study failed to analyze and discuss the differences in intervention effects among children of different genders due to the absent report of relevant information in the original literature. According to previous studies, the use of music and tools (props or equipment) in children’s physical activities seems to be an important factor affecting the results of intervention. Therefore, we conducted subgroup analysis according to the type of intervention (whether music or tools were used), the length and frequency of the intervention, and the subject of the evaluation of the intervention results.

#### 3.5.1 The effects of different types of DPA on preschoolers’ social skills score

Table 3 shows the effects of different types of DPA on preschoolers’ social skills testing score. We screened all the included studies, and according to the implementation of the original studies, we grouped them into four subgroups: with music and tools (*n* = 7), with music (*n* = 1), with tools (*n* = 3), and without music or tools (*n* = 1). Compared with the control group, it is noticeable that a significant and stable positive effect was identified in the interventions with both music and tools [SMD = 0.58, 95% CI (0.22, 0.94), *p* = 0.002, *I^2^* = 81% and Chi*^2^ p* < 0.0001], and those without music or tools [SMD = 0.66, 95% CI (0.14, 1.18), *p* = 0.01]. The interventions with only music [SMD = 0.24, 95% CI (−0.27, 0.75), *p* = 0.35] or tools [SMD = 0.97, 95% CI (−0.20, 2.14), *p* = 0.10] cannot improve the outcomes significantly. No significant difference was identified between the subgroups (*p* = 0.55). The results above indicated that the effects of the interventions incorporating both music and tools were more instrumental in enhancing children’s social skills, while that of those with only one strategy were less effective.

**Table 3:** The effects of different types of DPA on preschoolers’ social skills score.

Subgroup	Study	Heterogeneity		Test for overall effect		*SMD* [95% CI]	Difference between group *p*
		*p*	*I^2^*	*Z*	*p*		
Music & tools	7	<0.0001	81%	3.17	0.002	0.58 [0.22, 0.94]	0.55
Only music	1	–	–	0.93	0.35	0.24 [−0.27, 0.75]	
Only tools	3	<0.00001	92%	1.63	0.10	0.97 [−0.20, 2.14]	
None	1	–	–	2.48	0.01	0.66 [0.14, 1.18]	

#### 3.5.2 The effects of different intervention lengths and frequencies on preschoolers’ social skills score

Table 4 shows the effects of different DPA interventions based on the length and frequencies on social skills testing scores. We used a median split to determine a cutoff value of intervention length (12 weeks) and intervention frequency (2 times per week).

**Table 4:** Effects of different intervention length and frequencies on social skills.

Subgroups		Study	Heterogeneity		Test for overall effect		*SMD* [95% CI]	Difference between group *p*
			*p*	*I^2^*	*Z*	*p*		
Length (weeks)	<12	3	0.03	72%	1.71	0.09	0.46 [−0.07, 0.98]	0.02
	12	7	0.0001	78%	4.31	<0.0001	0.86 [0.47, 1.25]	
	>12	2	0.48	0%	0.61	0.54	0.10 [−0.23, 0.44]	
Frequency (times per week)	≤ 2	6	0.0004	78%	3.18	0.001	0.77 [0.30, 1.25]	0.67
	>2	5	<0.00001	86%	2.55	0.01	0.63 [0.15, 1.10]	

In terms of intervention length, effects of interventions less than 12 [SMD = 0.46, 95% CI (−0.07, 0.98), *p* = 0.09] and more than 12 [SMD = 0.10, 95% CI (−0.23, 0.44), *p* = 0.54] showed no significant difference between intervention and control groups, while interventions of 12 weeks [SMD = 0.86, 95% CI (0.47, 1.25), *p* < 0.0001] showed significant difference. Significant difference was identified between the intervention of less than 12 weeks, 12 weeks, and more than 12 weeks (*p* = 0.02).

In terms of the frequencies of the studies, effects of interventions with both 2 or less than 2 times per week [SMD = 0.77, 95% CI (0.30, 1.25), *p* = 0.001] and more than 2 times per week [SMD = 0.63, 95% CI (0.15, 1.10), *p* = 0.01] were identified with significant difference between the social skills testing score of intervention group and control group. No significant difference was identified between the intervention of ≤2 times per week and >2 times per week (*p* = 0.67).

#### 3.5.3 The effects of measurements completed by different subjects on preschoolers’ social skills score

Table 5 shows the effects of measurements completed by different subjects on social skills scores. All the included studies were identified as teacher-rating (*n* = 4), parent-rating (*n* = 7), and children-testing (*n* = 2). Compared with the control group, a significant effect was found in parent-rating subgroup [SMD = 0.73, 95% CI (0.45, 1.01), *p* < 0.00001], with moderate-to-high heterogeneity (*I^2^* = 60% and Chi*^2^ p* = 0.02), whereas insignificant effects were found in both teacher-rating group [SMD = 0.63, 95% CI (−0.10, 1.36), *p* = 0.09, *I^2^* = 90% and Chi*^2^ p* < 0.00001] and children-testing group [SMD = 0.50, 95% CI (−0.08, 1.08), *p* = 0.09, *I^2^* = 50% and Chi*^2^ p* = 0.16]. No significant difference was identified between the teachers-rating, parent-rating, and children-testing groups (*p* = 0.78).

**Table 5:** Effects of measurements completed by different subjects on social skills.

Subgroup	Study	Heterogeneity		Test for overall effect		*SMD* [95% CI]	Difference between group *p*
		*p*	*I^2^*	*Z*	*p*		
Teacher-rating	4	<0.00001	90%	1.70	0.09	0.63 [−0.10, 1.36]	0.78
Parents-rating	7	0.02	60%	5.04	<0.00001	0.73 [0.45, 1.01]	
Children-testing	2	0.16	50%	1.70	0.09	0.50 [−0.08, 1.08]	

### 3.6 Sensitivity analysis

**Figure 6:**
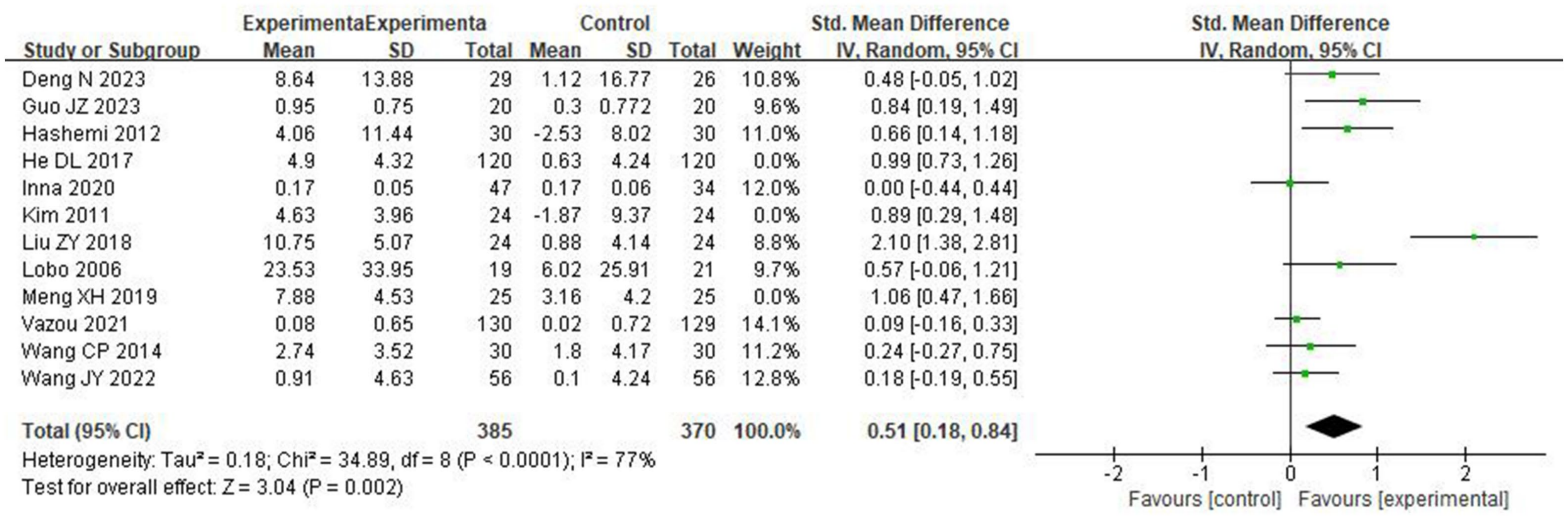
The result of sensitivity analysis.

A sensitivity analysis was performed, which excluded 3 poor-quality studies, for social skills, with the effect slightly decreased from 0.63 to 0.51. The result indicated the potential publication bias in the excluded studies (Figure 6).

To further strengthen the results above (SMD_SC_ = 0.63, SMD_EMO_ = 1.84), we conducted a sensitivity analysis examining a correlation of 0 or 0.9. The results showed that when the correlation was 0, the effects of DPA on the preschool children’s social skills (SMD = 0.46) and emotion (SMD = 1.34) remained significant. When the correlation was 0.9, the effects of DPA on the preschoolers’ social skills (SMD = 1.18) stayed significant, while that of emotion (SMD = 3.92) turned insignificant.

**Figure 7:**
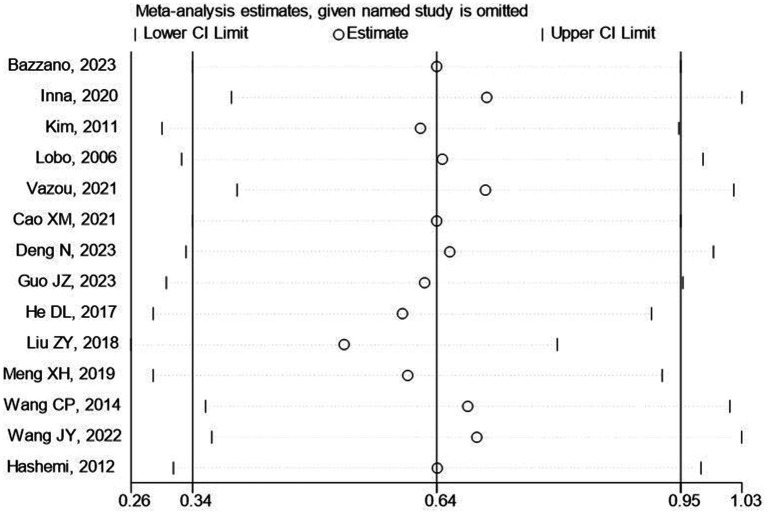
The influence of each study on the outcome of the meta-analysis (social skills).

**Figure 8:**
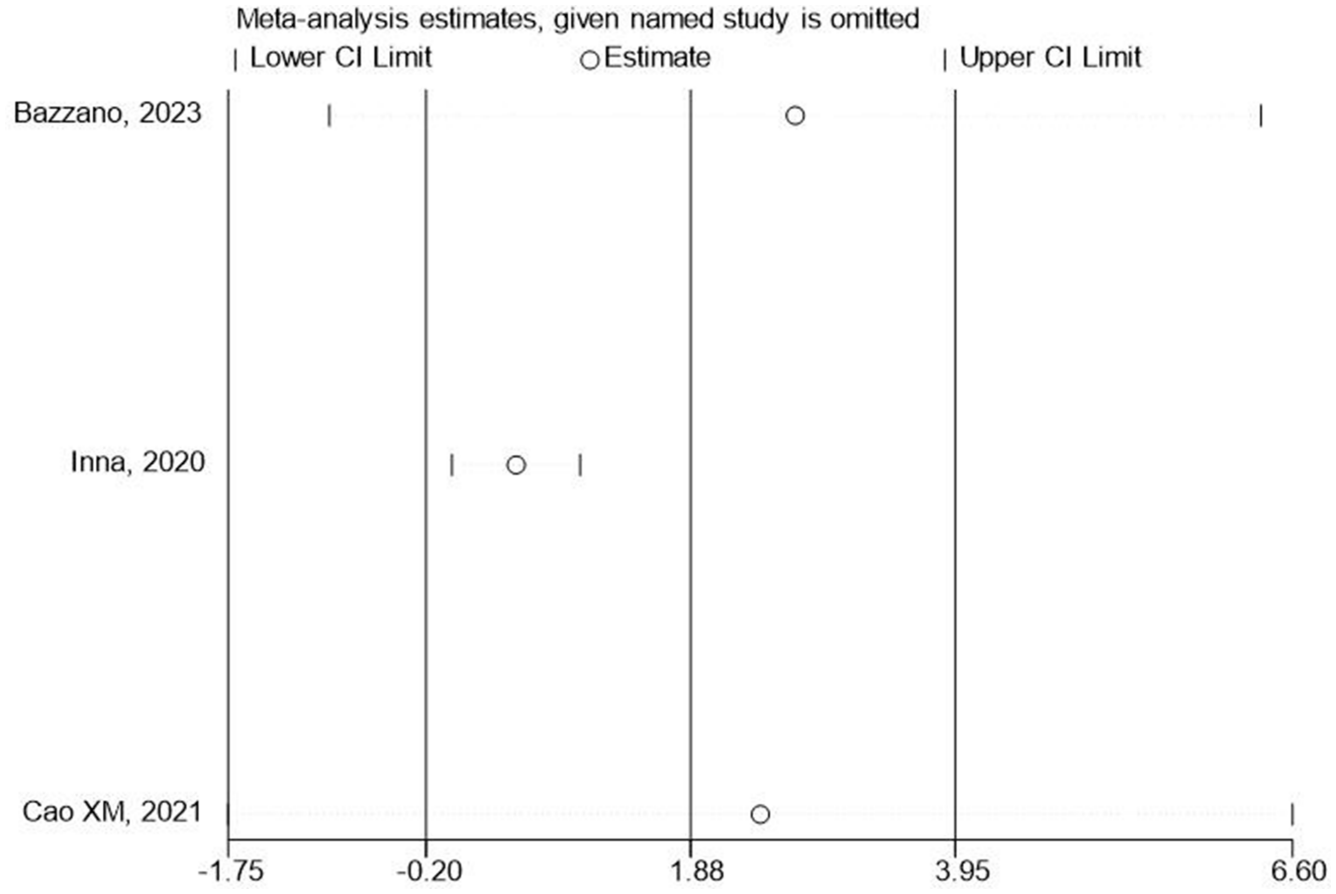
The influence of each study on the outcome of the meta-analysis (emotion).

We further conducted a leave-one-out analysis to evaluate the influence of each study on the outcome of meta-analysis (Figures 7, 8). The results showed that after removing a study, the effect size remained significant. All the results above indicated that the included literature’s stability was acceptable, and no specific study was significantly influential in the effect. In addition, we observed that when low-quality studies were excluded, the overall effect size was only slightly reduced, suggesting that the results of these low-quality studies did not have a significant impact on the overall effect size.

### 3.7 Publication bias

**Figure 9:**
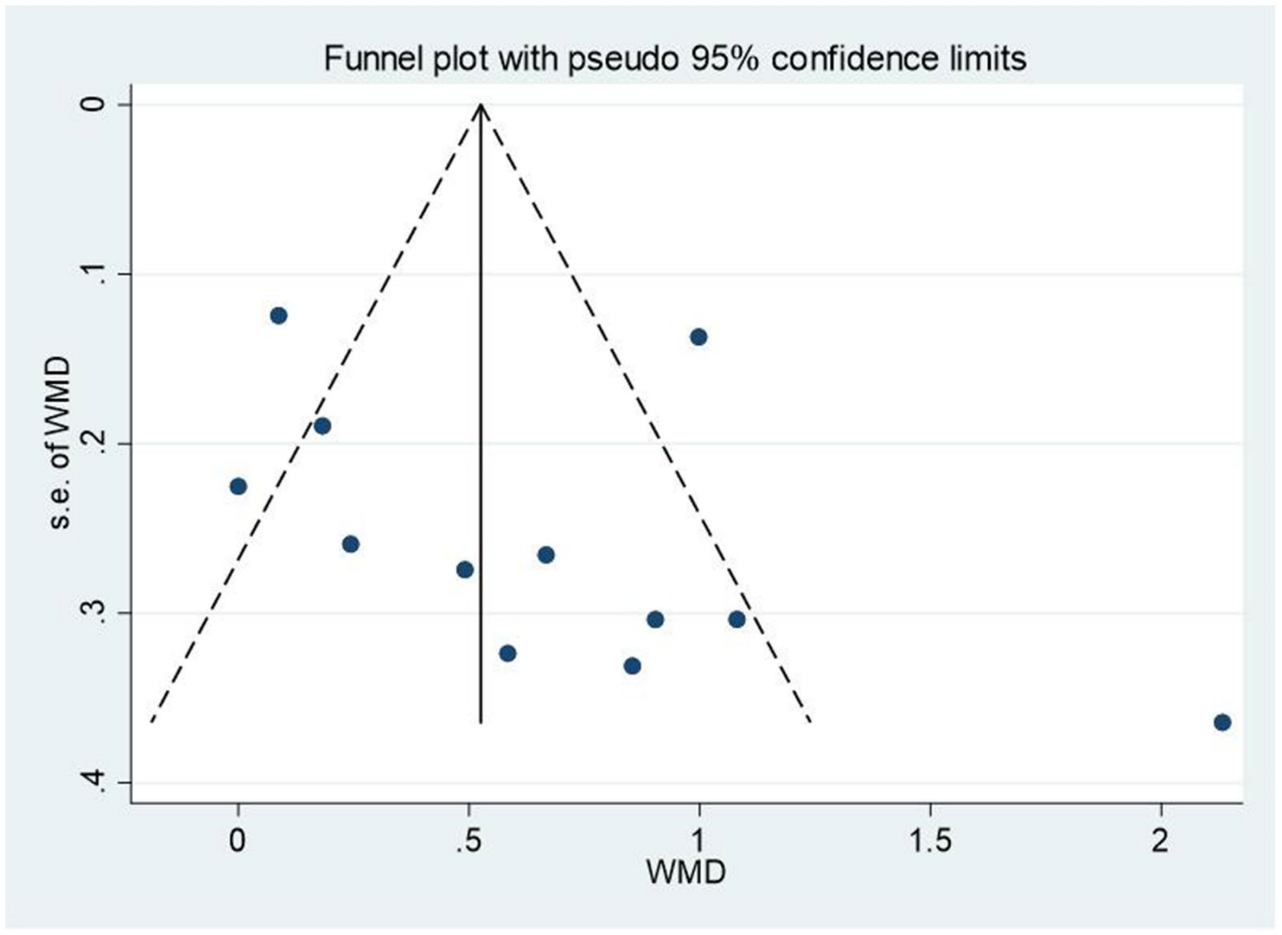
Funnel plot (social skills).

**Figure 10:**
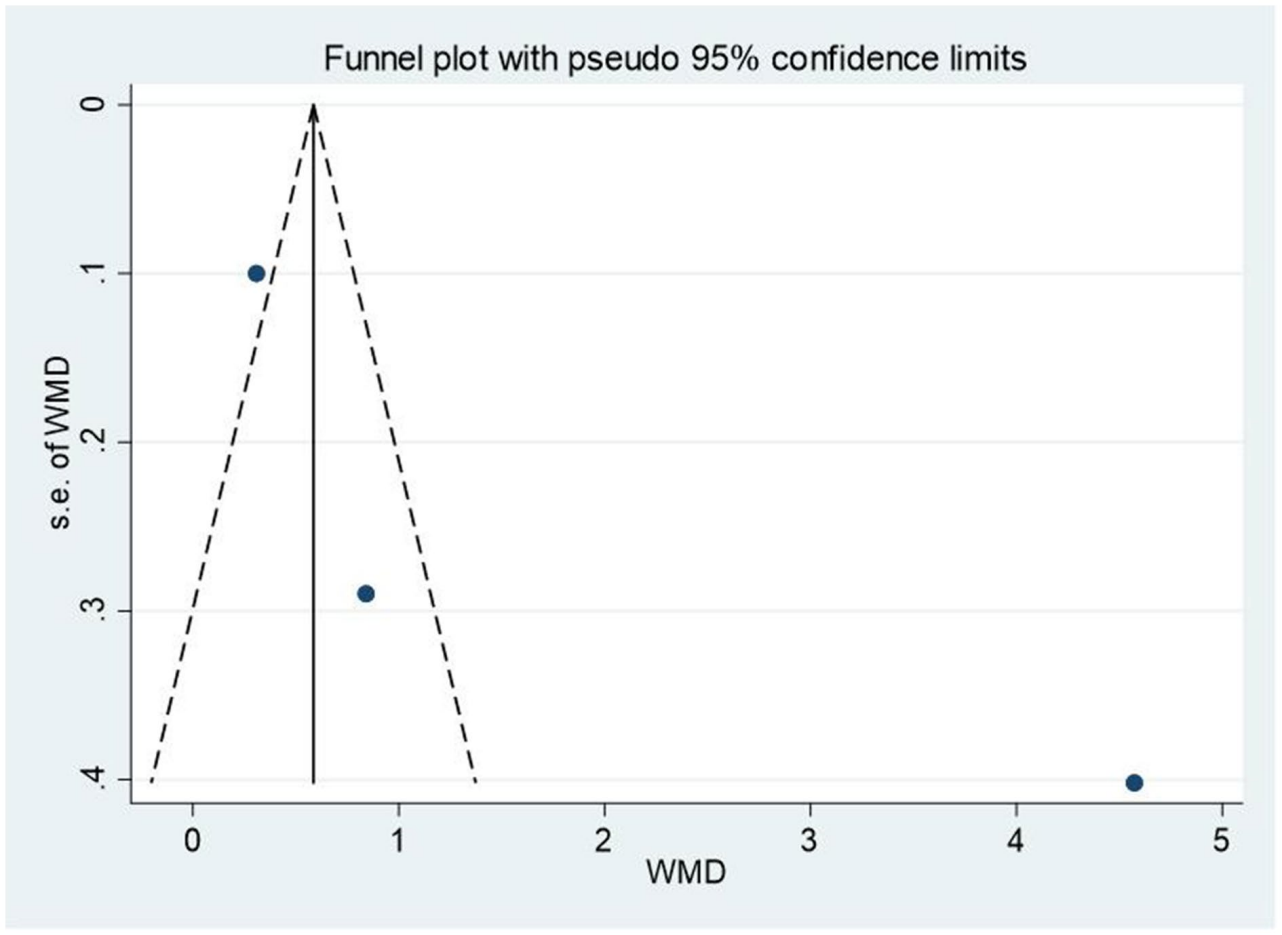
Funnel plot (emotion).

We used Egger’s test to assess the publication bias of all the included studies. The *p*-values were 0.207 (social skills) and 0.362 (emotion), implying no significant publication bias in this meta-analysis. To make the publication bias test results more intuitive, funnel plots were also supplemented, see Figures 9, 10.

## 4 Discussion

### 4.1 Summary of the findings

In the present meta-analysis study, we determined the effects of DPA on the social skills of preschool children, and further explored the variations when the intervention characteristics were different through the trails. Overall, there was a clear trend that DPA positively impacted the social skills [SMD = 0.63, 95%CI (0.33, 0.94)], and an insignificant trend that positively impacted emotional skill [SMD = 1.86, 95%CI (−0.20, 3.91)]. Generally speaking, when the effect size value is less than 0.2, it is considered a small effect; 0.5 indicates a medium effect; and values above 0.8 represent a large effect (Cohen, 1969). From the above results, it can be observed that the effect size of DPA on social skills was at a moderately high level, and the effect size was significant. Our findings were in accordance with those of a recent researches (Lu, 2023; Veiga et al., 2022; Veiga et al., 2017a; Veiga et al., 2017b). Different results also have been found in some of the researches, showing insignificant or negative results (Dong et al., 2019). The effectiveness of the studies is depended on the specific circumstances to a large extent, including the cultural background, participant characteristics, and the nature of the intervention itself, rather than the promise (Arslan et al., 2011; Maleki et al., 2019a, 2019b; Maleki et al., 2018). Therefore, meta-analysis is necessary for studies with diversity in implementation and outcomes. The region, the proportion of participants of varying genders and age groups, the preschool educators involved, the content of the interventions, the control measures employed, the dosage of the interventions, and the methods of measurement used in the studies that fulfilled our inclusion criteria varied from those of previous studies, thus resulting in distinct outcomes.

Scholars’ differing understandings of the concept and framework of social skills may be a key cause of heterogeneity. As mentioned in the introduction section, scholars currently have varying definitions of social skills. After reviewing all the original studies, it was found that Kats Gold et al. (2021) and Vazou and Mavilidi (2021) analyzed children’s social skills through cooperation, assertion, and self-control; Lobo and Winsler (2006) through social competence, internalizing behavior problems, and externalizing behavior problems; Hashemi et al. (2012) through social cooperation, social interaction, and social independence; He (2017), Meng (2019), and Wang C. (2022) through emotional integration, communication, and empathy; Liu (2018) through social initiative, prosocial behavior, verbal and non-verbal communication, and social impairment; Cao (2021) reflected children’s emotional adjustment through temper control, social assertiveness, and anxiety control; Bazzano et al. (2023) through initiative, self-control, attachment, and behavioral concerns; Deng D. (2023) through initiative and self-control; Guo (2023) through sports confidence, psychological regulation ability during exercise, enthusiasm for physical activity participation, awareness of physical activity rules, and interpersonal communication skills in sports; and Wang (2014) did not mention detailed information.

Although different understanding of social skills existed, there is a consensus among psychologists and educators that social skills are recognized important in individuals’ development trajectories, which could be observed during social exchange (American Occupational Therapy Association, 2014; Tal-Saban et al., 2021). Considering the complexities and intricacies in surroundings and children development, physical activity interventions have become a viable means of enhancing social skills development in early childhood education, which could not only promote physical development but also foster social interaction (Carson et al., 2019; Loukatari et al., 2019; Wang C., 2022). No doubt exists in the importance of physical activities in childhood period, and this is strongly supported by previous educational frameworks (Carson et al., 2017; Lopes et al., 2011; Melby et al., 2021).

Based on the background above, educators and pedagogists started to explore the way and content of physical activity intervention that could meet children’s developmental needs. An increasing number of scholars have designed physical activity interventions, and have proved them satisfied. Therefore, in our study, we focused on DPA, an effective intervention strategy specially designed for preschoolers under the consideration of the characteristics of preschoolers’ physical and mental development. DPA offers children supportive environments, which could meet their needs for developing social learning, cooperative behavior, and communication behavior (Berk and Trieber, 2009; McNally and Slutsky, 2020).

We can understand the results above through Social Ecological Systems Theory. Social Ecological Systems Theory indicates that the microsystem, as the immediate environment in which individuals directly participate, has a direct and significant influence on individual development. According to the original studies, some researchers have taken the significance of microsystems into account when designing DPA. For example, Kats Gold et al. (2021) has designed activities to sustain social games and conversational interactions, enhance story and instruction comprehension, and facilitate flexible adaptation to rule changes during role shifts in games. Kim et al. (2011) has created wrestling activities, Lobo and Winsler (2006) has designed solo and partner dance activities, Liu (2018) has developed group sports games, and Vazou and Mavilidi (2021) has emphasized the development of social awareness and interpersonal skills through active interactions with peers and teachers, as well as cooperation, sharing, and communication. These designs offer children more opportunities to interact with their environment, objects, and peers, thereby promoting better social development in children.

We have noticed that playgroup and play-based teamwork, affording participants opportunities to increase synchronization and coordination in body actions with the peers, communicate and express themselves, can foster their confidence and communication skills (Aldabas, 2022; Green et al., 2018; Rabinowitch and Meltzoff, 2017a; Tunçgenç and Cohen, 2018), and these are crucial for developing social interaction such as taking turns to play, negotiating conflicts, and cooperative play (Chatzipanteli and Adamakis, 2022; Tekyi-Arhin, 2023). However, in our study, we have also noticed that those interventions emphasizing independent tasks also achieved relatively good results. Some scholars view social skills as social initiative and behavioral control (Chen and French, 2008). This could explain why both intervention types can effectively promote children’s social skills development.

### 4.2 Subgroup analysis

We conducted subgroup analysis based on several variables, including the types, length, frequency, people who complete the test. Our results found that the effect of the both use of music and tools was better than either use music or tools alone. 12 weeks appeared to be the peak of the intervention effect, with no more than 12 weeks or less than 12 weeks being less effective than 12 weeks.

#### 4.2.1 The effect of different contents of interventions on social skills in preschool children

Based on the intervention implementation contents as reported in the original studies, we identified all the interventions into these characteristics: with/without music and with/without tools. Therefore, we defined four subgroups, specifically interventions with both music and tools, interventions with only music, interventions with only tools, and interventions without music or tools. The interventions implemented with music were identified more rhythmic and artistic, while those interventions without music were thought more sporty. And the interventions incorporating tools were considered more likely to promote participants’ manipulating skills. According to the results of the current study, interventions with both music and tools and interventions without music or tools showed significantly positive effects on children’s social skills, while the interventions with only music or tools could not increase the social skills.

The interesting findings from the subgroup analysis drew our focused attention. As for the interventions incorporating with music, such as creative dance, situationalized physical game, gymnastics, ball games in the included studies, there have been a number of previous studies suggesting the profound effects on social skills and social behaviors. Kirschner and Tomasello (2010) observed that children tended to choose to play with partners rather than by themselves, and they would like to prefer the partners with shared musical experience. This could potentially be attributed to the fact that the beats and rhythms may elicit a synchronization of interpersonal movement (Levitin et al., 2018), thereby serving as a catalyst for prosocial behavior in preschool-aged children (Cirelli et al., 2014; Rabinowitch and Meltzoff, 2017b; Tunçgenç and Cohen, 2018). Music itself is believed beneficial to modulate emotions and mood states, thus further cause influence on interpersonal behavior, communication, and cooperation (Cross, 2005), and an increasing number of researchers have been focused on potential links between music and social skills (Barrett et al., 2022; Finnigan and Starr, 2010; Good and Russo, 2016; Nousia, 2023). However, the results and the opinions of the studies were mixed. Positive effects of music to social skills have already been proved by a large number of scholars (Boal-Palheiros and Ilari, 2023; Chen et al., 2013; Deng D., 2023; Mansouri and Naseri, 2023; Maróti et al., 2019; Yum et al., 2020). Meanwhile, there were also negative outcomes. Schellenberg et al. (2015) pointed out that the positive effect may only exist in children who had low scores on the pretest. And Alemán et al. (2017) found no significant change in Venezuelan children’s social skills when his team implemented formal music education. Based on the varied outcomes and divergent opinions presented above, it is reasonable to conjecture that the integration of music and body movement can better stimulate the development of social skills rather than music itself. It has been discovered that under music-related contexts, humans usually couple their movements with the beat to express their feeling to music and imitate musical features through their body and body actions (Burger et al., 2012; 2013). This synchronization process, also named as entrainment, is considered especially effective to promote interpersonal similitude and coordination (Rabinowitch and Meltzoff, 2017a). Ilari et al. (2020) also found that this process may also increase the joy associated with making music with others, individual proclivities toward prosociality, and there may be even interactions between all these factors mentioned. And in the present study, among the DPA identified as playful activities with music, children were encouraged to listen to the music, dance to music, express their emotions, use imagination to act and imitate under play-based, music, and collective contexts. And under the contents with the tools, it could be more easy for children to interact with their peers through various tools, such as balls, ropes, and elastic bands. According to the implementations reported by the authors of the original studies, children will be required to play a series of games through different types of balls (including mini-football, mini-basketball, table tennis etc.), and the games will be organized in various situationalized way, such as working as teams delivering the ‘goods’ from one spot to another, protecting the flag (preventing the flag from being taken by others) (Wang C., 2022). These kind of interventions could stimulate the body movements of the preschoolers, which was proved beneficial for increasing children’s social interaction, and further promote social skills (Hartanto et al., 2021; Tersi and Matsouka, 2020).

It is noteworthy that our findings reveal a substantial enhancement in preschoolers’ social skills scores, even in the absence of musical or instrument-based interventions. This was also in accordance with some of the previous studies (Dereli-İman, 2014; Özbey and Köyceğiz, 2019). The effectiveness and the importance of DPA without music or tools should not be denied and ignored simply due to the profound meaning and effect of music and tools on social skills. The educators and the scholars should better understand that it is not the music and tools themselves that cause the improvements in children’s social skills, but the body movements and the physical activities that the music and tools induce. Furthermore, the lack of included studies may also attribute to this result.

#### 4.2.2 The effect of interventions with different length and frequencies on social skills in preschool children

Based on the results above, we noticed differences between the studies with different length, and all the outcomes were significantly effective regardless of the frequency of intervention. Significantly positive outcomes were only identified in those interventions lasted for 12 weeks, and conversely, those interventions lasted shorter than or longer than 12 weeks showed insignificant effects. One possible explanation is that unlike free play, children participating in DPA are first required to learn the movements before they can interact and cooperate with their peers. These movements generally consist of various motor skills that take time and effort for young children to master. Therefore, the early effect of DPA interventions can be lower than that of usual activity. After some time, the children get increasingly familiar with the movements, and gradually, they start to have the capacity to interact with their peers, express their thoughts and emotions, try to understand others, and cooperate with their peers while carrying out the physical activities. Thus, within a certain period of time, the effect of DPA starts to appear. As for the insignificant results of those interventions that lasted longer than 12 weeks, we conjectured that the reason may be due to the gradual development of mental fatigue through the repetitive content and similar strategy (Junfei et al., 2024; Wylie et al., 2020). Regarding the intervention frequencies, the outcomes demonstrated notable and consistent positive results, irrespective of the specific frequency employed. However, this does not prove that growth in young children’s social skills is fully correlated with DPA intervention. According to Social Ecological Systems Theory and Dynamic Systems Theory, young children develop certain social skills in the kindergarten setting by being with their peers or playing outdoor (Hinkley et al., 2018). In early childhood research, we cannot isolate young children growing up in a solitary setting. Thus, it is difficult to determine a direct and exact link between intervention and the improvement of young children’s social skills.

In our study, we failed to further explore the different effect of DPA on different age groups due to the lack of information reported in the original studies. The rapid development of children’s body and mind signifies that notable disparities in their social skills may emerge between pre-test and post-test, regardless of the any intervening measures. This could be explored and discussed in further studies. The findings above should be interpreted with caution. As in the above results, while a high degree of heterogeneity could be seen in the overall social skills scores, emotional scores, and the subgroups, Egger’s asymmetry test indicates a symmetrical distribution of effect sizes and a low risk of publication bias.

### 4.3 Limitation

First, to guarantee the quality of the studies included in this meta-analysis, we have confined our selection to studies written in Chinese, English, or Korean. This decision excludes conference papers, gray literature, unpublished studies, and those written in other languages, which may potentially introduce publication bias and language bias, thereby failing to encompass the full spectrum of relevant research. Second, while measuring solely social skills may not provide a comprehensive assessment, combining measurements of both social skills and their subdomains would yield a more exhaustive understanding. However, due to the scarcity of studies that meet our criteria and encompass all subdomains of social skills, we were unable to incorporate such studies into our analysis. Third, despite our efforts, we have identified heterogeneity among the studies included. Notably, subgroup analysis did not fully mitigate this heterogeneity. The implementation of empirical studies in preschool children is considered with difficulties regardless of the regions, and the fact does not always in accordance with promise. Therefore, the results should be interpreted with caution. Additionally, numerous methodological shortcomings have been discerned, including incomplete reporting of participants’ gender, lack of allocation concealment, and insufficient use of blinding techniques. Also, studies with different study designs were included. These weaknesses cast doubt on the validity of our findings. Furthermore, it is important to note that children aged 3 to 6 years from the original studies were eligible for inclusion in this meta-analysis. Consequently, sources of heterogeneity may stem from variations in subject conditions, research designs, and implementation methodologies. Also, there may be interactive effects among these factors that further contribute to the observed heterogeneity.

In our study, one of the notable and interesting findings is that we noticed different results from different raters. The effects of the teacher-rating and children-testing studies were not significant, while those of the parent-rating studies showed significance. Our results are in accordance with previous research. Tal-Saban et al. (2021) have noticed that parents reported better social skills and fewer behavioral problems. The differences in multi-subjective evaluation were also identified in other studies related to children development (Ren, 2022), and parents were more likely to give higher scores when they were asked to evaluate their own child (Su et al., 2016). One possible reason is that children may behave different in kindergarten and at home due to the distinguishes between the environment, atmosphere, care-takers and their coping styles, etc. (Fengmeng, 2016; Maleki et al., 2019a, 2019b). Researches have shown that interaction contextual factors, evaluator subjective factors, and the access to information of the evaluators can all influence the results (He, 2012). Although the results are different, according to our results, we can still hypothesize higher consistency between teacher ratings and children test results, with parent ratings tending to be higher. This may also partially explain the source of heterogeneity in the included studies.

A further limitation of the present meta-analysis concerns the reliance on proxy reports. Children aged 3–6 years generally lack the cognitive and linguistic capacities to provide valid self-reports of their own social behavior. Consequently, researchers typically depend on proxy measures, such as parent or teacher reports, to assess children’s social skills. However, proxy reporting may introduce systematic bias, because the report reflects the observer’s perspective, possibly shaped by their expectations, beliefs, and subjective considerations, rather than the child’s actual social behavior. Proxy measures are known to be vulnerable to several biases, including recall bias and social desirability bias (Arts et al., 2022). In the context of child health and behavior assessments, proxy reports often manifest poor agreement with children’s self-reports, particularly in psychosocial domains: meta-analytic evidence indicates that inter-rater reliability between self- and proxy-reported health-related constructs—such as ‘feeling worried, sad, or unhappy’—tends to be low (Khanna et al., 2022). This suggests that while proxy reporting may capture observable behaviors, it may inadequately reflect internal or emotional states, which are central to social skill development. Thus, although proxy reporting is a pragmatic necessity in studies involving preschool-aged children, investigators must acknowledge that these data may not fully represent children’s authentic social skills. Future research should aim to minimize reliance on proxy measures where feasible—through alternative assessment methods developed for this age group—or at least systematically account for such biases in study design and interpretation.

Cultural factors may significantly influence the effectiveness of DPA, yet this aspect was not thoroughly explored in our study. One reason is that the primary goal of this study was to analyze the impact of DPA on young children’s social skills, and the inclusion criteria were based primarily on the original data. These studies did not address the cultural aspects of the interventions. However, we found that in Kim et al. (2011), where the DPA content was traditional Korean sports games, adapting physical activities to fit the local cultural context helped young children adapt more quickly to the activities. This adaptation could enhance their ability to apply the acquired social skills in daily social interactions and also assist evaluators in scoring based on their understanding of the local culture.

Furthermore, regarding missing data, one study used an intention-to-treat (ITT) analysis (Guo, 2023), five studies used a per-protocol (PP) analysis (Cao, 2021; Deng D., 2023; Kats Gold et al., 2021; Lobo and Winsler, 2006; Vazou and Mavilidi, 2021), while eight studies did not reported missing data and either the way to deal with it (Bazzano et al., 2023; Kim et al., 2011; Hashemi et al., 2012; He, 2017; Liu, 2018; Meng, 2019; Wang, 2014; Wang C., 2022). Different ways of dealing with missing data could result in different degrees of bias. Intention-to-treat analysis, applying full analysis set (FAS), could avoid the bias due to the lost of the participants or participants’ disobey, providing higher consistencies with the baseline. However, per-protocol analysis require the researchers only include the valid data, therefore, offering a higher effect size of the intervention (Porta et al., 2007). This should be noticed and considered while making conclusions.

### 4.4 Implications for practice and research

First, at present, in the physical activity intervention for preschool children, the specific activity types, frequency and length of intervention, and measurements of outcome indicators were many, which can affect the authenticity and reliability of the research results to a certain extent. Therefore, to improve the methodological quality, more randomized controlled trials with rigorous study designs and larger sample sizes are required to strengthen the control of bias from the aspects of the use of blinding and allocation concealment. Second, a comparative analysis should be conducted to evaluate the impacts of various types of physical activities and differing intervention doses, in order to ascertain the optimal and most appropriate content and scope of DPA intervention. Third, to enhance the depth of exploration into DPA intervention, future research endeavors should encompass an examination of brain architecture, cognitive capabilities, and the underlying processes that govern them. Additionally, when confronted with absent data, an ITT analysis is recommended as a means to mitigate potential biases, as it is viewed as a more prudent approach. Meanwhile, reporting the results of both ITT analysis and PP analysis could help the readers better understand the bias. Moreover, as discrepancies may arise from different raters’ assessments, it is recommended to collect the outcome from diverse perspective in order to enhance the reliability of the original research findings.

## 5 Conclusion

DPA exhibit a positive influence on the social skills and emotional skill of preschool children. However, given the current evidence base, the interpretation of these outcomes should be interpreted with caution. Nevertheless, DPA can serve as a pivotal factor in enhancing preschoolers’ social abilities, fostering a foundation for an active lifestyle, and facilitating the attainment of various physiological, social, and cognitive health advantages. It is plausible to hypothesize that the incorporation of music into DPA may yield even more pronounced effects. Consequently, there is a pressing need for future high-caliber research endeavors to ascertain the definitive effectiveness of DPA in children by exploring optimal program designs, dose–response relationships, and long-term sustainability.
